# Cost-effectiveness of a transdiagnostic psychotherapy program for youth with common mental health problems

**DOI:** 10.1186/s12913-022-08187-9

**Published:** 2022-06-24

**Authors:** Rasmus Trap Wolf, Pia Jeppesen, Mette Maria Agner Pedersen, Louise Berg Puggaard, Mikael Thastum, Niels Bilenberg, Per Hove Thomsen, Wendy K. Silverman, Kerstin Jessica Plessen, Simon-Peter Neumer, Christoph U. Correll, Anne Katrine Pagsberg, Dorte Gyrd-Hansen

**Affiliations:** 1 Child and Adolescent Mental Health Center, Mental Health Services – Capital Region of Denmark, Copenhagen, Denmark; 2 Danish Centre for Health Economics, Department of Public Health, University of Southern Denmark, Odense, Denmark; 3 Department of Clinical Medicine, Faculty of Health and Medical Sciences, University of Copenhagen, Copenhagen, Denmark; 4 Department of Child and Adolescent Psychiatry, Copenhagen University Hospital – Psychiatry Region Zealand, Smedegade 16, 4000 Roskilde, Denmark; 5 Centre for the Psychological Treatment of Children and Adolescents, Department of Psychology and Behavioural Sciences, Aarhus BSS, Aarhus University, Aarhus, Denmark; 6 Department for Child and Adolescent Psychiatry, Mental Health Services in the Region of Southern Denmark, Odense, Denmark; 7 Institute of Clinical Medicine, University of Southern Denmark, Odense, Denmark; 8 Research Center at the Department for Child- and Adolescent Psychiatry, Aarhus University Hospital, Skejby, Denmark; 9 Institute of Clinical Medicine, Aarhus University, Aarhus, Denmark; 10 Anxiety and Mood Disorders Program, Yale Child Study Center, Yale School of Medicine, New Haven, CT USA; 11 Division of Child and Adolescent Psychiatry, Department of Psychiatry, Lausanne University Hospital CHUV, Lausanne, Switzerland; 12 Centre for Child and Adolescent Mental Health, Oslo, Norway; 13 The Arctic University of Norway, Centre for Child and Youth Mental Health and Child Welfare, North Norway (RKBU North), Tromsø, Norway; 14 Department of Psychiatry and Molecular Medicine, Donald and Barbara Zucker School of Medicine at Hofstra/Northwell, Hempstead, NY USA; 15 Department of Psychiatry, The Zucker Hillside Hospital, Glen Oaks, NY USA; 16 Center for Psychiatric Neuroscience, Feinstein Institute for Medical Research, Manhasset, NY USA; 17 Department of Child and Adolescent Psychiatry, Charité Universitätsmedizin, Berlin, Germany

**Keywords:** Children, Adolescents, Anxiety, Depression, Behavioral problems, Cognitive behavioral intervention, Transdiagnostic, Cost-effectiveness, Informant, Preference weights

## Abstract

**Objectives:**

Our objective was to evaluate the cost-effectiveness of the transdiagnostic psychotherapy program Mind My Mind (MMM) for youth with common mental health problems using a cost-utility analysis (CUA) framework and data from a randomized controlled trial. Furthermore, we analyzed the impact of the choice of informant for both quality-of-life reporting and preference weights on the Incremental Cost-Effectiveness Ratio (ICER).

**Methods:**

A total of 396 school-aged (6–16 years) youth took part in the 6-month trial carried out in Denmark. CUAs were carried out for the trial period and four one-year extrapolation scenarios. Costs were based on a combination of budget and self-reported costs. Youths and parents were asked to report on the youth’s quality-of-life three times during the trial using the Child Health Utility 9D (CHU9D). Parental-reported CHU9D was used in the base case together with preference weights of a youth population. Analyses using self-reported CHU9D and preference weights of an adult population were also carried out.

**Results:**

The analysis of the trial period resulted in an ICER of €170,465. The analyses of the one-year scenarios resulted in ICERs between €23,653 and €50,480. The ICER increased by 24% and 71% compared to the base case when using self-reported CHU9D and adult preference weights, respectively.

**Conclusion:**

The MMM intervention has the potential to be cost-effective, but the ICER is dependent on the duration of the treatment effects. Results varied significantly with the choice of informant and the choice of preference weights indicating that both factors should be considered when assessing CUA involving youth.

**Supplementary Information:**

The online version contains supplementary material available at 10.1186/s12913-022-08187-9.

## Background

Mental health disorders affect many children and adolescents below the age of 18 years (herein referred to as youths) [1–3]. The most common mental health disorders among youth; mild to moderate symptoms of anxiety, depression, and disruptive behavior disorders have an increased risk of adverse adult outcomes [4]. Several studies have found promising effects of cognitive-behavioral therapy (CBT) programs for these disorders [5–12]. Still, there is limited access to evidence-based programs. Mind My Mind (MMM) is a transdiagnostic CBT-program for indicated prevention and early treatment of anxiety, depression, and behavioral disturbances below the threshold for psychiatric referral. It was designed to address the problem of limited access by providing a feasible program for large-scale implementation. MMM has recently demonstrated superiority over Management As Usual (MAU) in a pragmatic, multi-site, and randomized controlled trial (RCT) [13].

Despite the abundance of studies investigating interventions for common mental health disorders among youth, evidence of the cost-effectiveness of indicated prevention and interventions is still limited [14–20]. CBT programs typically involve a considerable cost due to the amount of time delivered by the therapists. The budget impact is likely to be part of the explanation for the limited access to evidence-based programs. This calls for cost-utility analyses (CUA) of evidence-based interventions to inform decision makers.

Analyzing the cost-utility of interventions for youth presents several challenging aspects. While the methodology and the guidelines for estimating Quality-Adjusted Life Years (QALY) in adult populations are well developed, there is less clear guidance for youth populations [21]. Therefore, a recent review, therefore, warrants further empirical evidence on the valuation of youth-specific preference-based measures [22]. In a mental health setting, the choice of informant is especially important, as experiences and preferences across youths and parents are found to vary [23]. Similarly, the preferences of the different quality-of-life health states vary in the mental dimensions when comparing preferences weights derived from youth and adult populations [24].

In this study, we analyzed the cost-effectiveness of the MMM intervention using a CUA framework. We also present how the CUA results were impacted by choice of informant (patients or parents) and the choice of preference weights (tariffs) used in the QALY estimation. The CUA was based on data collected in the RCT of MMM versus MAU with a trial period of 26 weeks [13]. MMM aims at treating current symptoms and preventing the development of severe mental disorders. To derive an accurate estimate of the long-term effect of an intervention, it is recommended that health effects and costs are extrapolated for as long as they are assumed to differ between the compared interventions. This is often done using decision-analytic modeling that incorporates data from external sources [25]. However, there is limited data to inform modeling in this case. Few studies of CBT programs have long term follow-up. For those that have long term follow-up, beneficial and statistically significant effects are found up to 12 months after the end of treatment [5–12]. Several cohort studies have also shown associations between psychopathological outcomes and later risk of a severe mental disorder [26, 27]. However, the associations have not been investigated in an experimental design, which makes intervention-based improvements uncertain. The limited data and uncertainty about the long-term effects lead us to analyze different possible scenarios of a one-year extrapolation period. This means that we primarily analyzed the treatment effect of MMM, and not the potential preventive effects.

## Methods

### Trial design

Full details of the trial design are described in separate papers [13, 28]. In brief, the MMM trial was a pragmatic open-label randomized controlled trial carried out in Denmark in a local municipality setting. Participants were randomly allocated on an individual level 1:1 via independent, blinded, computer-generated allocation sequences with variable and unknown block sizes, stratified by geographical region, age-group (6–10 years or 11–16 years) and their top-problem as defined by the children together with their parents (anxiety, depressive symptoms, behavioral problems). The sample size for the trial was based on the effectiveness outcomes of the trial and not HRQOL or costs outcomes. Data used in the present analyses were collected at baseline, end-of-treatment (week 18), and follow-up (week 26). The trial was approved by the local scientific ethics committee (Journal nr.:H-17011408). The study was registered at ClinicalTrials.gov (Identifier NCT03535805).

#### Participants

A total of 396 (197 randomized to MMM and 199 to MAU) youth aged 6–16 years were enrolled in the trial. A two-stage standardized visitation based on parental referral was implemented in the Educational-psychological advisory service in the four participating municipalities to identify study participants. Further description of the visitation procedure is available elsewhere [28]. Eligibility criteria were: 1) Age between 6 to 16 years, 2) being in 0-9^th^ grade, 3) having a parent-reported score on The Strength and Difficulties Questionnaire (SDQ) above a cut-off based on the top 10% of mental health problems in the general age-matched Danish population [29], and 4) having anxiety, depressive symptoms, or behavioral problems as the top-problem based on the standardized assessments in the visitation procedure. Youth with a prior diagnosis of any developmental or mental disorder, including autism spectrum disorder, attention-deficit/hyperactivity disorder, psychotic disorder, eating disorder, severe obsessive–compulsive disorder, repeated self-harm, alcohol or psychoactive drug abuse, or with signs of intellectual disability were not eligible for the trial. Further description of the participants is available elsewhere [13, 28].

### Intervention

The MMM intervention is a transdiagnostic, manualized, modular CBT-program comprising 9–13 individual sessions plus a booster-session targeting anxiety, depression and/or behavioral problems. All treatment was delivered by trained local psychologists from the Educational-psychological advisory service in the four municipalities. The treatment was supervised by psychologists from the regional Child and Adolescent Mental Health Services (CAMHS). For further description of the training see Table SM1 in the supplementary material. Youth randomized to MMM were not eligible for other therapeutic interventions offered by the municipalities during the treatment and follow-up period. They could seek help from other health care professionals, e.g. general practitioners (GP). If the mental health condition of the youth progressed to the level at which specialized treatment was indicated according to standard guidelines, they were referred to CAMHS at any point during the trial [13].

### Comparator

Youth and parents in the MAU group were offered two care-coordination visits to help coordinate usual care in the municipality. These visits were not a standard part of usual care in the municipalities but were introduced in the trial period to make sure the services provided under MAU were salient to the parents and to reduce the risk for attrition. The treatment offered in MAU varied considerably from no intervention to individual and group therapy, and parental training. Psychologists trained in MMM did not conduct care-coordination visits or provide any kind of therapy to the MAU group. The youth in the MAU group could also seek help from other health care professionals or be referred to CAMHS.

### Perspective and time horizon

The CUAs were conducted from an extended health sector perspective and included the costs of all interventions directed toward the individual youth’s mental health problems no matter the provider of services. No data was available on indirect interventions like e.g. teacher support and broader class-based preventive interventions.

**Fig. 1:**
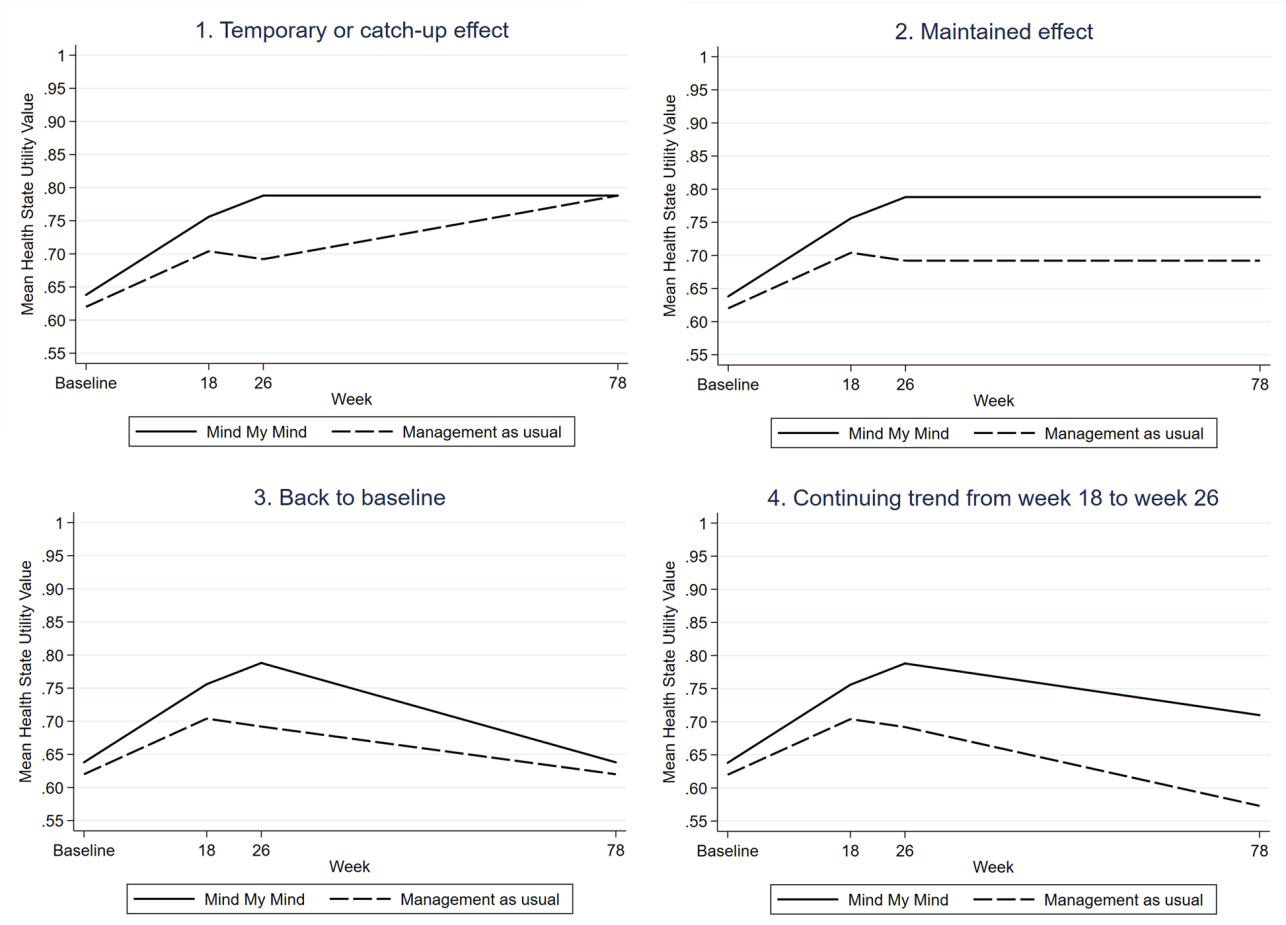
Mean health state utility for the four scenario extrapolations

The base case analysis had a 26-week time horizon consistent with the trial length. As outlined in the Background section the lack of data limits the possibilities of carrying out formal decision analytic modelling. Instead four one-year extrapolations scenarios of the development in HRQOL were analyzed using individual-level data (see Fig. 1): 1) Temporary or catch-up effect, 2) Maintained effect, 3) Back to baseline, 4) Continuing trend from end-of-treatment to follow-up. In scenario 1 we assumed that the effect of MMM was either temporary or there was a catch-up effect from the worst performing group so that the MMM and the MAU group would end up having the same average health state utility value (HSUV) one year after the last observed time point (week 26). The incremental QALY-gain is the same independently of which group is assumed to be steady and which group is assumed to change in mean HSUV, thus, the scenarios were analyzed as one. In scenario 2 we assumed that both groups would have the same HSUV one year after follow-up as they had at follow-up. In scenario 3 we assumed that the trial period only had a temporary effect, so that one year after follow-up, all youth would have the same HSUV as they had at baseline. In scenario 4 we assumed that the trend in the period from end-of-treatment (week 18) to follow-up in which only a booster-session was given, would continue for one year. For scenario 4 we also assumed the highest and lowest HSUV at the extrapolation timepoint to be the highest and lowest observed HSUV. Given that there are no direct intervention driven costs after the trial period had ended, we assumed no group difference in costs during the one-year extrapolation in all analyses of the scenarios, as we have no evidence to the contrary.

### Costs

All costs were estimated at the individual participant level. All data on the Mind My Mind intervention was based on collected administration data during the trial. Data on individual MAU and other health care use were collected by standardized parent-reported questionnaires at end-of-treatment and follow-up. Table SM1 in the supplementary material provides information on the specific cost components, assumptions, sources of unit costs, and cost estimates. All costs are presented in Euros (€). We converted the Danish kroner (DKK) costs using the currency rate DKK100 = €13.38.

#### Mind My Mind

The intervention costs comprise the cost of training the psychologists and the supervisors, the costs of materials for the intervention, and the salaries of the psychologists and supervisors. The training costs were divided equally among youth to estimate individual-level costs. As the training of the psychologists can be considered an investment, the full return of the training will not be achieved within the trial period. This was addressed in a sensitivity analysis.

#### Management as usual

The MAU costs comprise the cost of the care-coordination visits and costs of individual and group therapy, and parent psychoeducation provided by the municipality.

#### Other health care use

The other costs included in the analyses comprise health care use related to mental health problems including visits to the GP, pediatrician, CAMHS, and private-sector psychologists.

### Effect

The health effects are expressed in QALY. To calculate QALYs, the youth’s health states must be determined, and each state must be given a utility. In the base case, health states were defined using parent’s answers to The Child Health Utility 9-Dimension (CHU9D), and the utilities associated with each health state were derived from tariffs based on preferences of youth aged 11–17 years.

CHU9D is a generic preference-based health-related quality of life (HRQOL) measure constructed for use in youth. CHU9D has nine items with five levels of severity representing nine dimensions of HRQOL: Worried, Sad, Pain, Tired, Annoyed, Schoolwork/homework, Sleep, Daily routine, and Activities [30]. CHU9D is validated in mental health settings [31, 32]. Parents and youth both completed the CHU9D questionnaire online at baseline, end-of-treatment and a follow-up. For consistency with the primary clinical outcome of the trial [13], and due to the lower proportion of missing data, parents’ responses were used in the base case.

A validation of CHU9D in a mental health setting has been done using blinded data from the MMM trial [32], but there are no Danish tariffs for CHU9D. In the study, two sets of tariffs were investigated: 1) preferences of Australians aged 11–17 years from the general population (*N* = 1,982) [33], 2) preferences of the adult general population in the United Kingdom (*N* = 300) [34]. The validation study showed that CHU9D is an appropriate HRQOL measure for use in mental health trials, and that the preference weights generated from the adolescent population resulted in larger mean differences between groups with different levels of mental health problems [32]. Based on these findings and the sample size of the populations the tariffs of Australians aged 11–17 years were used in the present base case.

### Missing data

Descriptive analyses were carried out to determine how to handle missing outcome data following a guide for handling missing data in cost-effectiveness analyses conducted within randomized trials [35]. There were no missing baseline CHU9D values: there were 57 (14%) missing at end-of-treatment, and 63 (16%) at follow-up. For the self-reported cost components there were 59 (15%) missing at end-of-treatment and 63 (16%) at follow-up. In total, 74 (19%) had a missing value at any timepoint. There was a higher proportion of missing values in the MAU group compared to the MMM group for both utilities and costs (74 (24%) versus 48 (18%) missing at any time point). Logistic regression models found the treatment group to be the only statistically significant predictor of missing values when investigating baseline utility, sex, age-group, region, and top problem (anxiety, depression, or behavioral difficulties). Based on the findings, it was assumed that the probability of data being missing was independent of unobserved characteristics, hence, the data were treated as being missing at random within each group.

Multiple-imputation using chained-equations was performed on utility score level and cost-component level. Due to the nature of utility scores and costs, predictive mean matching was used in the imputation. Twenty imputations were performed based on the proportion of missing data [36]. Imputations were performed separately for each trial group. To be consistent with the analysis of the clinical outcomes [13], the imputation models included baseline utility, sex, age-group, region, and top problem. After imputation, incremental QALYs and costs were calculated as the mean of the incremental QALY estimates and incremental costs estimates, respectively, generated in each imputed dataset following Rubin’s rule [37]. The distributions of the observed and imputed values confirmed the validity of the imputation procedure. Complete case analysis was conducted, confirming the robustness of the imputations. The analysis is available in the supplementary material Table SM2.

### Data analysis and results

The RCT found that the MMM intervention leads to significantly better clinical outcomes than MAU with a group difference in SDQ Impact score decrease of 1.10 (95% CI 0.75–0.145) [13]. We thus expected positive incremental QALY results. The results of the CUAs are therefore presented as the Incremental Cost-Effectiveness Ratio (ICER):

$$ICER=\frac{\mathrm{\Delta Cost}}{\mathrm{\Delta Effect}}=\frac{{\mathrm{Cost}}_{\mathrm{MMM}}-{\mathrm{Cost}}_{\mathrm{MAU}}}{{\mathrm{QALY}}_{\mathrm{MMM}}-{\mathrm{QALY}}_{\mathrm{MAU}}}$$

Estimates of costs and effects were calculated using Seemingly Unrelated Regression models [38]. Adjustment for baseline utility were included in the estimation of QALYs to account for the imbalance between treatment groups [39]. P-values below 0.05 were considered statistically significant. All analyses were performed in STATA-15 [40].

### Uncertainty

#### Sample uncertainty

Sample uncertainty was examined using non-parametric bootstrapping with 10,000 iterations. Bootstrap samples of 396 were drawn from each of the 20 multiple imputed datasets, and the difference in net benefit between the groups was calculated for each bootstrap sample given different thresholds [41]. The proportion of bootstrap samples in which the net benefit is positive represents the probability that the treatment is cost-effective for each imputed dataset. The probability is then averaged across all the imputed datasets to construct cost-effectiveness acceptability curves.

#### Sensitivity analyses

The psychologist’s training can be considered an investment in which the full return is based on the number of youths each psychologist treated. The restricted trial-period makes the number of youths each psychologist treated lower than the full potential. We therefore conducted sensitivity analyses, in which we assumed the number of treated youths per psychologist to be the highest number one psychologist treated within the trial-period.

The impact of the choice of the informant who evaluates the youth’s health state (the parent or the youth) and the tariffs that are subsequently applied (youth’s or adult’s) to estimate QALYs were explored by conducting a sensitivity analysis using youth’s responses to CHU9D, and another sensitivity analysis using the tariffs derived from a general adult population.

Furthermore, sensitivity analyses of the one-year extrapolation period were performed by estimating ICERs for alternative time periods (6 and 18 months) in the four extrapolation scenarios presented in Perspective and time horizon section.

## Results

### Cost

MMM was estimated to cost €3,471 on average per youth. The cost of the different components of MMM, including the training of psychologists, is available in the supplementary material Table SM1. The cost of training a psychologist was €9,178 and €12,119 for training a supervisor. The cost of the training was divided equally among the youth randomized to MMM. This made the number of treated youths per psychologist in the trial period the most determinant factor of the total cost of the intervention. On average each psychologist treated 8.2 youth, with the highest number being 20 youth for one psychologist. If all trained psychologists treated 20 youths, the average cost would be €2,476.

The costs of MMM, MAU, and other health care utilization are presented in Table 1. The 199 youths allocated to MAU received an average of 1.6 coordinating visits. 198 (95%) received at least one coordinating visit. The mean incremental costs for MMM were €2,981. MAU and private-sector psychologists were the primary sources of costs difference besides MMM. There were no other statistically significant differences in other health care utilization.

**Table 1:** Costs

	Mind My Mind (*n* = 197)		Management as usual (*n* = 199)	
	Units, Mean ± SE	Cost €, Mean ± SE	Units, Mean ± SE	Cost €, Mean ± SE
**Mind My Mind**	1	3,471 ± 19		
**Management as usual**				
Care-coordination visits			1.6 ± 0.0	111 ± 3
Individual sessions with a psychologist			1.7 ± 0.3	117 ± 20
Group therapy with a psychologist			0.8 ± 0.3	9 ± 3
Parent psychoeducation			0.1 ± 0.1	9 ± 5
MAU Total			247 ± 22	
**Other health care**				
General practitioner	0.5 ± 0.1	35 ± 11	0.7 ± 0.3	48 ± 25
Pediatrician	0.4 ± 0.2	27 ± 11	0.3 ± 0.1	17 ± 8
Child and Adolescent Mental Health Services	0.3 ± 0.1	82 ± 30	0.4 ± 0.1	99 ± 32
Private-sector psychologist	0.9 ± 0.3	107 ± 34	2.8 ± 0.7	329 ± 85
Other health care total	252 ± 63		493 ± 101	
**Total costs**	3,722 ± 64		741 ± 105	
**Incremental costs**	2,981 (95% CI: 2,731–3,251)			

Costs are presented as mean (standard error (SE)) in Euros and are based on the intention-to-treat population from 20 imputed datasets. 95% Confidence interval of total costs is derived from 10,000 bootstrap replications from 20 imputed datasets using the standard normal method

### Effects

MMM had a non-statistically significant higher baseline utility and statistically significant higher utility at both end-of-treatment and follow-up. MMM gained 0.360 QALY in the period and MAU 0.337 QALY. After adjusting for baseline utility, the incremental QALYs were estimated to be 0.017 for MMM. Table 2 presents the utilities for the observed mean values.

**Table 2:** Health-related quality of life and quality-adjusted life years

	MMM (*n* = 197) Mean ± SE	MAU (*n* = 199) Mean ± SE
**Health-related quality of life**		
Baseline	0.638 ± 0.015	0.620 ± 0.015
End-of-treatment (week 18)	0.756 ± 0.015	0.704 ± 0.017
Follow-up (week 26)	0.788 ± 0.014	0.692 ± 0.018
**QALY**	0.360 ± 0.006	0.337 ± 0.007
**Incremental QALYs**		
Unadjusted	0.024 ± 0.009	
Adjusted for baseline	0.017 (95% CI: 0.006–0.029)	

Values presented are the mean (standard error (SE)) of the intention-to-treat population from 20 imputed datasets. Adjusted incremental Quality Adjusted Life years (QALY) are adjusted for baseline utility. The confidence intervals (CIs) were derived from 10,000 bootstrap replications from 20 imputed datasets using the standard normal method

The four one-year extrapolation scenarios are illustrated in Fig. 1 with the mean HSUV for the two groups at the three observed time points and at the extrapolated time point (week 78) one year after the last observed time point (week 26). The counter-intuitive development in scenario 4 with decreasing mean HSUV was due to a higher number of youths reaching the HSUV ceiling level of 1 during the extrapolation period than the number reaching the floor level of 0.053.

All four scenarios resulted in statistically significant higher incremental QALYs compared with the base case. Adjusted for baseline utility the mean incremental QALY were 0.059 (95% CI: 0.023–0.101) for scenario 1, 0.104 (95% CI: 0.056–0.151) for scenario 2, 0.061 (95% CI:0.032–0.089) for scenario 3, and 0.126 (95% CI: 0.065–0.186) for scenario 4.

### Cost-effectiveness

The ICER was estimated to be €170,465 per QALY gained for the restricted base case analysis.

**Fig. 2:**
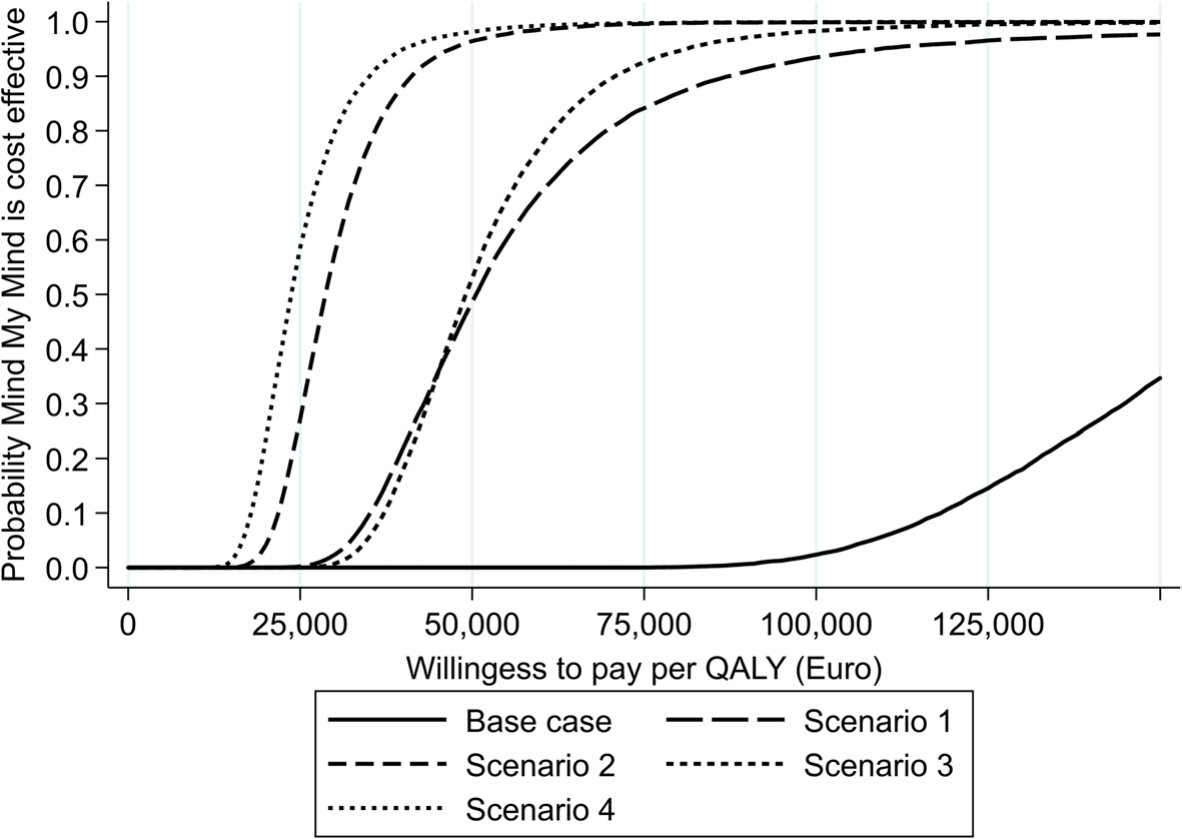
Cost-effectiveness acceptability curves

The analyses of the four one-year scenario extrapolations resulted in the following ICERs: €50,480 per QALY gained for scenario 1, €28,659 per QALY gained for scenario 2, €49,069 per QALY gained for scenario 3, and €23,653 per QALY gained for scenario 4. The cost-effectiveness acceptability curves (CEAC) are shown in Fig. 2.

### Sensitivity analyses

The ICER was €114,713 when assuming each psychologist would be able to treat 20 youth each instead of the average of 8.2 that was observed in the trial-period.

To analyze the sensitivity of the duration of the scenario extrapolations on results, the ICERs were calculated using the same assumptions but changing the period from 1 year to 6 and 18 months, respectively. For the 6 month scenario, the ICERs ranged from €41,245 to €77,894. For the 18 months scenarios the ICERs range from €16,659 to €37,339. All ICERs and CEACs are available in the supplementary materials Table SM3 and Fig. SM1.

The base case ICER is based on QALYs estimated using parent-reported CHU9D and tariffs based on the preferences of a youth population. Table 3 shows the results of two separate analyses. One in which the self-reported CHU9D is used in the QALY estimation instead of the parent-reported, and another analysis in which the tariffs are based on preferences of an adult population rather than on a youth population. The two analyses resulted in non-statistically significantly lower incremental QALYs compared to the base case. The ICER was €40,292 (24%) higher than the base case when using self-reported CHU9D and €121,451 (71%) higher when using the tariffs based on an adult population. CEACs are available in the supplementary materials Fig. SM2.

**Table 3:** Analyses using self-reported CHU9D and tariffs from an adult population

	**Self-reported CHU9D** Tariffs based on preferences of a youth population		**Parent-reported CHU9D** Tariffs based on preferences of an adult population	
	MMM (*n* = 197) mean ± se	MAU (*n* = 199) mean ± se	MMM (*n* = 197) mean ± se	MAU (*n* = 199) mean ± se
Baseline	0.642 ± 0.016	0.631 ± 0.016	0.806 ± 0.009	0.802 ± 0.008
End-of-treatment (week 18)	0.728 ± 0.016	0.684 ± 0.018	0.873 ± 0.008	0.844 ± 0.009
Follow-up (week 26)	0.769 ± 0.015	0.708 ± 0.186	0.886 ± 0.008	0.839 ± 0.009
QALY	0.352 ± 0.007	0.335 ± 0.007	0.426 ± 0.003	0.414 ± 0.004
Incremental QALYs				
Unadjusted	0.017 ± 0.009		0.012 ± 0.005	
Adjusted for baseline	0.014 (95% CI: 0.002–0.027)		0.010 (95% CI: 0.004–0.016)	
ICER	€210,757 /QALY gained		€291,916 /QALY gained	

Values presented are the mean (standard error (SE)) of the intention-to-treat population from 20 imputed datasets. Adjusted incremental quality adjusted life years (QALY) are adjusted for baseline utility. The confidence intervals (CIs) were derived from 10,000 bootstrap replications from 20 imputed datasets using the standard normal method

*CHU9D* Child Health Utility 9D, *ICER* Incremental Cost-Effectiveness Ratio

## Discussion

The ICER found in the base case with the time horizon restricted to the trial time period was €170,465. There is no explicit willingness-to-pay in Denmark. The estimate is, however, higher than what would be considered standard willingness to pay per QALY. For the scenarios with a continuing effect (scenarios 2 and 4), the ICERs were below €30,000, and the MMM intervention had a 90% probability of being cost-effective at a willingness to pay per QALY gained of €41,000 (see Fig. 2). For scenarios 1 and 3, assuming temporary effects, the ICERs were estimated to be around €50,000. The sensitivity analyses addressing the assumption of a one-year extrapolation period showed that all scenarios resulted in ICERs below €40,000 when an 18-month extrapolation period was assumed. In contrast only scenarios 2 and 4 had ICERs below €50,000, when a 6-month extrapolation period was assumed.

In light of the large proportion of youth suffering from common mental health disorders and the promising effects of CBT-programs, it is surprising that there is limited access to evidence-based programs. MAU was chosen as a comparator in the trial as no evidence-based programs are widely accessible for youths with common mental health problems. Coordination visits were offered in order to ensure that all parents in the MAU group were made aware of the services the municipalities offered under MAU. This enhanced the MAU and incurred a minor cost given the time used by professional from the municipality, furthermore, the enhancement might have had a positive effect for the MAU group which would limit the relative effect of MMM. Most evidence-based CBT-programs are problem-specific and only relevant for a part of the population with common mental health problems. Instead, the transdiagnostic and modular approach in MMM made it possible to offer treatment to a large and heterogeneous population of youth with emotional and behavioral problems. A considerable part of the costs of delivering MMM is associated with the training of psychologists and supervisors. The high training costs support the choice of offering the transdiagnostic MMM instead of traditional CBT-programs as this avoids parallel training costs for three single-disorder programs to target the same group of youth. The sensitivity analysis showed that the cost estimate decreased by 33% when assuming each psychologist would treat 20 youth instead of the average of 8.2 in the trial-period. The high costs were partly due to the training being held at a hotel, including the stay costs. If the same level of training can be delivered in a different setup, for example online, there could potentially be significant savings, but this remain uncertain.

Due to the short observation period, we only considered the immediate effects of the MMM intervention. As outlined in the Background section it is hypothesized that the observed positive effect of MMM will generate long-term preventive effects by lowering the risk of future severe mental disorders [13]. If this is the case, MMM may be considerably more cost-effective than our results suggest, both due to the health effects and also due to the potential cost savings, as access to CAMHS is associated with substantial health care costs [29].

In this study, we applied a health care sector perspective. A previous review has, however, shown that the majority of costs of mental disorders among youth are in other sectors, primarily the education and social sectors [42]. Furthermore, we have not included the parents’ costs and benefits in this study. Positive spillover effects for parents are often observed in interventions that benefit their offspring [43]. We lack data on these broader costs and benefits, but given the positive effects of MMM compared to MAU, it is possible that a cost-effectiveness analysis with a societal perspective would generate lower ICERs than observed in this study.

The HRQOL at baseline in our trial was similar irrespective of whether reported by the youth or the parent. However, at end-of-treatment and follow-up, the youth rated their HRQOL lower than their parents did in both groups. The self-reported CHU9D resulted in a 24% higher ICER. We could only identify one other CUA that reports CHU9D for both informants [16]. In that study, surprisingly, almost no difference in the QALY-gain was found when comparing across informant types. Our findings align better with previous findings from a review of other preference-based HRQOL instruments used in youth, that concludes that youth and parental responses are not interchangeable [44].

The impact of using the adult tariffs from the UK (rather than the youth tariffs from Australia) was even greater making the ICER 71% higher. This difference may be driven by the youth population placing greater weight on the mental health dimension of CHU9D compared to adults [24]. However, there are other methodological differences between the two tariffs besides the age and nationality of the samples they are based on, which could also explain some of the difference. The youth tariffs are derived using discrete-choice experiments and a smaller sample time-trade-off experiment with young adults to anchor the tariffs [33], while the adult tariffs are derived using a standard gamble experiment [34].

For health technology assessment agencies that aim for comparability across cost-effectiveness studies, our finding that QALY estimates are highly sensitive to the choice of informant with respect to both HRQOL reporting and tariffs, warrants attention. Since these choices are not trivial, they should be further scrutinized in developing guidelines for CUA of interventions for youth.

## Conclusion

Whether the MMM intervention is cost-effective depends on the duration of treatment effects and the intervention’s preventive impact. Restricting the analysis period to the trial period resulted in a cost per QALY-gained well above the standard willingness to pay. Applying four different one-year extrapolation scenarios resulted in ICERs within a range that is likely to be considered cost-effective. An additional important source of variation in ICER estimates was the choice of an informant for HRQOL and tariffs. This is a methodological aspect that warrants further attention.

Decisions on introducing MMM should consider the many uncertainties. It may be difficult to generate more precise information on the long-term effects of MMM via RCTs. However, future registry based studies can contribute to advancing understanding of the preventive effects of MMM.

## Supplementary Information

**Additional file 1: Table SM1.** Cost components, assumptions, and sources of unit costs. **Table SM2.** Complete cases analysis. **Table SM3.** Sensitivity scenarios. **Fig. SM1.** Cost Effectiveness Acceptability Curves for 6- and 18-months scenarios. **Fig. SM2.** Cost Effectiveness Acceptability Curves for sensitivity analyses results with self-reported CHU9D and adult tariffs.

## Data Availability

The pseudonymous data can be made available from 6 months after the publication date of this Article, and with no end date. Proposals for the use of data and requests for access should be directed to pia.jeppesen@regionh.dk. To gain access, researchers will need to sign a data access agreement with the Research Unit of the Child and Adolescent Mental Health Centre—Capital Region of Denmark, Copenhagen, Denmark.

## Abbreviations

CAMHS: Child and Adolescent Mental Health Services
CHU9D: Child Health Utility 9D
youth: Children and adolescents
CBT: Cognitive-behavioral therapy
CEAC: Cost-effectiveness acceptability curves
CUA: Cost-utility analysis
HRQOL: Health-related quality of life
HSUV: Health state utility value
MAU: Management As Usual
MMM: Mind My Mind
QALY: Quality Adjusted Life Years
RCT: Randomized controlled trial
ICER: The Incremental Cost Effectiveness Ratio
SDQ: The Strength and Difficulties Questionnaire
