# Supplementary material for: Cost-effectiveness of a transdiagnostic psychotherapy program for youth with common mental health problems
Source: BMC Health Serv Res. 2022 Jun 24;22:819. doi: 10.1186/s12913-022-08187-9 (PMC9229821; doi:10.1186/s12913-022-08187-9)
Supplement: Supplementary file 1 — Additional file 1: Table SM1. Cost components, assumptions, and sources of unit costs. Table SM2. Complete cases analysis. Table SM3. Sensitivity scenarios. Fig. SM1. Cost Effectiveness Acceptability Curves for 6- and 18-months scenarios. Fig. SM2. Cost Effectiveness Acceptability Curves for sensitivity analyses results with self-reported CHU9D and adult tariffs. [file 12913_2022_8187_MOESM1_ESM.docx]

**Supplementary Materials**

**Table SM1: Cost components, assumptions, and sources of unit costs**

| **Cost component** | **Assumptions** | **Source of unit costs** | **Cost estimate** |
| --- | --- | --- | --- |
| Salaries | The hourly salary of psychologist from the psychological school services and psychologist from the Child and Adolescent Mental Health Services (CAMHS) were separately based on the average salary of psychologists employed in the Danish municipalities and psychologists employed in the Danish regions. An effective working load of 1,600 hours a year were assumed. | Office for Municipal and Regional Wage Data ^45^ | €47 per hour for psychologist from Educational-psychological advisory service.  €47 per hour for supervisors from CAMHS. |
| **Mind My Mind** | | | |
| Training of psychologist from the psychological school services | It was assumed that all psychologist received the full training package. This was not the case as some psychologist did not participate in the whole trial period. The costs are based on the primary training course where the majority of the psychologists got their training. The cost of training one psychologist was calculated as the average costs of the following plus the salary of the psychologist to participate in the training.   - A one-week training course: Four-night stay at a hotel plus salary of teachers. - Three two-day training courses: One-night stay at a hotel plus salary of teachers. - Three one-day methodology courses: Seven participants had local courses. Costs only comprised the salary of teachers. | Cost from the trial budget + salaries | €9,178 per trained psychologist. |
| Training of supervisor psychologist from the Child and Adolescent Mental Health Services | The same assumptions made for the training of the psychologist from the psychological school services apply for the supervisors, as the supervisor underwent the same training together with psychologist. The supervisors further had the following:   - Three one-day courses: Five participants at a hotel setting plus salary of teachers. | Cost from the trial budget + salaries | €12,119 per trained supervisor. |
| Mind My Mind sessions | The actual number of MMM sessions the youth received were recorded on individual-level. The psychologists were allocated three hours per session for the first three youth they treated, hereafter they were allocated two hours per session. | (see salaries) |  |
| Materials | Materials included the printed Mind My Mind Manual, small games and accessories for the sessions, and a tablet for recording the sessions. It is assumed the materials will last for the treatment of 100 youth. | Estimated by trial manager | €1,338 |
| Supervision | The number of hours of supervision was based on the trial budget. 75% was to be delivered one-to-one online and 25% in groups in each of the four participating municipalities. As it was not possible to decide which youth were discussed when in the supervision, the total number of supervision hours were divided equally among the 197 youth randomized to Mind My Mind. That resulted in an average of 6.1 hours of individual supervision and 4.1 hours of group supervision per youth. | (see salaries) | €792 per youth randomized to Mind My Mind. |
| **Management as usual** | | | |
| Care-coordination visits | The actual number of care-coordination visits was recorded on individual-level. An hour consultation and half an hour preparation for each meeting was assumed. | (see salaries) |  |
| Individual therapy | The number of sessions with individual therapy provided by the municipality was based on self-reporting by the parents at end-of-treatment (week 18) and at follow-up (week 26). Each session was assumed to have a duration of one hour and require half an hour preparation. | (see salaries) |  |
| Group therapy | The number of sessions with group therapy provided by the municipality was based on self-reporting by the parents at end-of-treatment (week 18) and at follow-up (week 26). Each session was assumed to have a duration of one hour and require half an hour preparation. It was assumed that six youth take part in group therapy. | (see salaries) |  |
| Parental training | The number of sessions with parental training provided by the municipality was based on self-reporting by the parents at end-of-treatment (week 18) and at follow-up (week 26). Each session was assumed to have a duration of one hour and require half an hour preparation. | (see salaries) |  |
| **Other health care use** | | | |
| General practitioner (GP) | For visits at the GP it was assumed that therapy was provided by the GP. | The collective agreement between the Danish Regions and the Danish association of general practitioners ^46^. | €74 per visit. |
| Pediatrician | All visits to pedestrians were assumed to be standard consultations. | The Danish Health Data Authority ^47^. | €66 per visit. |
| Child and Adolescent Mental Health Services (CAMHS) | The cost of a visit to CAMHS a based on the standard tariff of an out-patient visit to CAMHS. | The Danish Health Data Authority ^48^. | €247 per visit. |
| Private-sector psychologist | The prices at private psychologist are not regulated. Prices for subsidized private psychologist are, however, publicly available. The costs of private-sector psychologist were therefore based on these prices. | The Danish Regions ^49^. | €117 per visit. |

**Table SM 2: Complete cases analysis**

|  |  |
| --- | --- |
| Incremental costs | €2,978 ±140 |
| Incremental QALY adjusted for baseline | 0.018 ±0.006 |
| Incremental Cost Effectiveness Ratio | €164,416 /QALY gained |

Note: Costs and QALY presented as mean ± standard error. Mind My Mind n=170, Management as usual n=150.

**Table SM 3: Sensitivity scenarios**

|  | Incremental Cost Effectiveness Ratio |
| --- | --- |
| 6 months extrapolation | |
| Scenario 1 | €77,894 |
| Scenario 2 | €49,069 |
| Scenario 3 | €76,202 |
| Scenario 4 | €41,245 |
| 18 months extrapolation | |
| Scenario 1 | €37,339 |
| Scenario 2 | €20,240 |
| Scenario 3 | €36,184 |
| Scenario 4 | €16,659 |

Note: Values presented are based on the intention-to-treat population from 20 imputed datasets. Incremental costs are the same as in base case.

**Fig SM1: Cost Effectiveness Acceptability Curves for six- and 18-months scenarios**


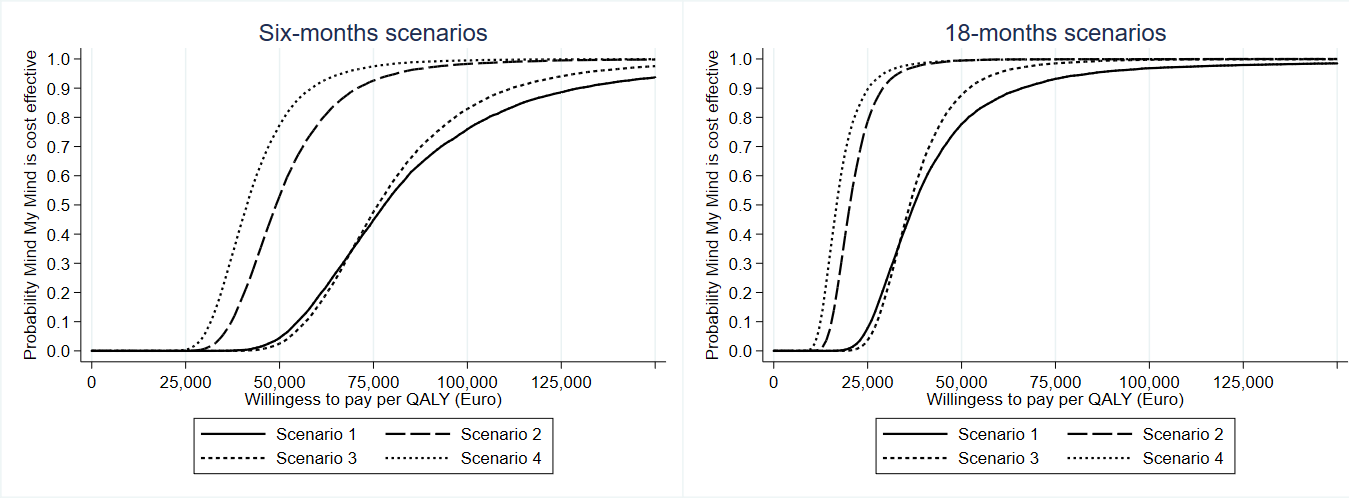


**Fig SM2: Cost Effectiveness Acceptability Curves for sensitivity analyses results with self-reported CHU9D and adult tariffs**
